# Efficacy and Side Effects of Drugs Commonly Used for the Treatment of Lower Urinary Tract Symptoms Associated With Benign Prostatic Hyperplasia

**DOI:** 10.3389/fphar.2020.00658

**Published:** 2020-05-08

**Authors:** Zhao-Jun Yu, Hai-Lan Yan, Fang-Hua Xu, Hai-Chao Chao, Lei-Hong Deng, Xiang-Da Xu, Jian-Biao Huang, Tao Zeng

**Affiliations:** ^1^Department of Urology, The Second Affiliated Hospital of Nanchang University, Nanchang, China; ^2^Medical Department of Graduate School, Nanchang University, Nanchang, China; ^3^Department of Clinical Medicine, Xi'an Jiao Tong University Health Science Center, Xi'an, China; ^4^Department of Pathology, Jiangxi Provincial People's Hospital Affiliated to Nanchang University, Nanchang, China; ^5^Institute of Clinical Medicine, Jiangxi Provincial People's Hospital Affiliated to Nanchang University, Nanchang, China; ^6^Department of Ultrasound, The First Affiliated Hospital of Nanchang University, Nanchang, China

**Keywords:** lower urinary tract symptoms, benign prostatic hyperplasia, efficacy, side effect, drug

## Abstract

Benign prostatic hyperplasia (BPH) is the most common benign disease of the prostate gland and is caused by benign hyperplasia of the smooth muscle cells and stromal cells in this important gland. BPH is also the most common disease underlying lower urinary tract symptoms (LUTS). The incidence of BPH increases with age and affects more than half of all men 50 years or older. BPH mainly exerts effects on urinary function and can seriously reduce a patient's quality of life. At present, treatment for BPH aims primarily to improve the quality of life and reduce the risk of BPH-related complications. Pharmacological therapy is recommended for moderate-to-severe cases of LUTS that are suggestive of BPH. A range of drugs is currently available to treat this condition, including α1-adrenoceptor antagonists, 5α-reductase inhibitors (5-ARIs), phosphodiesterase type 5 inhibitors (PDE5Is), muscarinic receptor antagonists (MRAs), β3-adrenoceptor agonists, and plant extracts. Of these, the most commonly used drugs in the clinic are α1-adrenoceptor antagonists, 5-ARIs, and combination therapy. However, these drugs exert their effects *via* various mechanisms and are associated with adverse reactions. The purpose of this review is to provide current comprehensive perspectives on the mechanisms of action, efficacy, and adverse reactions associated with the drugs most commonly used for the treatment of BPH.

## Introduction

Benign prostatic hyperplasia (BPH) is one of the most common benign diseases of the urinary system in middle-aged and elderly men. The prostate is an almond-shaped organ located at the junction of the bladder and the urethra, and comprises the central zone, peripheral zone, and transitional zone. BPH is an irregular benign hyperplasia of smooth muscle cells and stromal cells in the transitional area of the prostate gland that compresses the urethra, and can result in a series of lower urinary tract symptoms (LUTS) (Lee et al., 1997). LUTS involves voiding symptoms, such as a sensation of incomplete bladder emptying, straining to void, urinary hesitancy, and a weak urinary stream. Other problems include storage symptoms, such as nocturia, frequent urination, dysuria, and urinary urgency. Of these symptoms, the most common is nocturia; this is followed by a weak urinary stream, a sensation of incomplete bladder emptying, urgency, and urinary incontinence (Homma et al., 2017; Langan, 2019). In addition to LUTS, BPH can cause further complications including sexual dysfunction, urinary incontinence, renal insufficiency, urinary tract infection, and acute urinary retention (AUR) (Juliao et al., 2012). Although BPH is not lethal, it can seriously affect a patient's quality of life (QOL) by causing anxiety, sleep disorders, and sexual disharmony.

The morbidity of BPH can exceed 50% in men older than 50 years and can exceed 80% in men who are 70 years of age and above; evidence clearly shows that the morbidity of BPH increases with age (Egan, 2016). In addition, BPH can be induced by a range of risk factors, including obesity, hyperlipidemia, type 2 diabetes, and certain hormonal disorders (Calogero et al., 2019; Yoo et al., 2019). Interestingly, recent a meta-analysis found that the periodontal disease may be positively associated with an increased risk of BPH (Wu et al., 2019). In addition, research has shown that the long-term excessive consumption of alcohol, diuretics, dicyclomine, and caffeine may also contribute to LUTS, although moderate levels of alcohol intake have been shown to reduce the incidence of BPH (Bradley et al., 2017). At present, BPH can be treated by both non-pharmacological and pharmacological treatments. Over the past few decades, a wide variety of drugs have been developed to prevent the progression of BPH and improve the QOL. Many BPH patients achieve satisfactory treatment outcomes after receiving such treatments. This review focuses on the mechanism of actions, therapeutic efficacy, and major side effects of the drugs that are commonly used to treat BPH and shows newer perspectives about these drugs.

## Methodology

We conducted an extensive literature search of PubMed for articles relating to the drugs used to treat BPH or LUTS. We also searched several official websites for specific drug information or guidelines relating to BPH or LUTS, including the United States Food and Drug Administration (FDA), the European Association of Urology (EAU), and the American Urological Association (AUA). The following search terms were used: benign prostatic hyperplasia, BPH, lower urinary tract symptoms, LUTS, drug, treatment, therapy, safety, efficacy, side effect, adverse reaction, mechanism, α1-adrenergic antagonists, α1-blockers, 5α-reductase inhibitors (5-ARIs), muscarinic receptor antagonists (MRAs), β3-adrenoceptor agonists, beta-3 agonists, phosphodiesterase5 inhibitors (PDE5Is), plant extracts, and combination therapy. We also used the names of specific drugs as search terms, including tamsulosin, silodosin, doxazosin, alfuzosin, terazosin, tadalafil, mirabegron, tolterodine, and fesoterodine.

## Current Therapies for BPH

At present, the primary goals of BPH treatment are to ameliorate LUTS, improve QOL, inhibit disease progression, and reduce complications. The treatment of BPH involves three different stages: watchful waiting, drug therapy, and surgical treatment (Kim et al., 2016). Watchful waiting is recommended by the AUA for patients in whom QOL has not been influenced by mild LUTS; this strategy includes the implementation of dietary changes, exercise, education, and regular review (Mcvary et al., 2011). However, for patients with severe LUTS, watchful waiting is often ineffective and may delay optimal treatment; such patients need to be administered appropriate medication. A range of drugs are currently available for the treatment of BPH, including α1-blockers, 5-ARIs, MRAs, PDE5Is, β3-adrenoceptor agonists, and plant extracts. Of these, the most commonly used drugs are α1-blockers, 5-ARIs, and a combination treatment featuring both α1-blockers and 5-ARIs (Kim et al., 2016).

Several potential drugs have recently been developed for the treatment of BPH, including RS17503 (an α-adrenoceptor antagonist), RONO2 (an endogenous organic nitrate), SR49050 (an antagonist of the vasopressin receptor subtype V1A), and BXL-628 (a vitamin D3 agonist). RS-17503 has a high affinity for α1a-adrenoceptor and is a promising drug for the treatment of BPH. RONO2 has been used in several *in vitro* experiments and has been shown to increase Q-max and mean voided urine volume while reducing postvoid residual volume (PVR), and International Prostate Symptom Score (IPSS). Because vasopressin plays a physiological role in the contraction of the smooth musculature in both the prostate and urethra, there is considerable speculation that SR49059, a drug that targets the vasopressin receptor, might represent a potential candidate for the treatment of BPH (Uckert et al., 2019). However, as yet, none of these drugs have been tested in clinical studies, except for BXL-628. The clinical efficacy of BXL-628 in the treatment of BPH was not satisfactory; consequently, the clinical development of this drug was terminated (Colli et al., 2006). None of the other agents listed herein have been approved by the FDA for the treatment of BPH.

According to AUA guidelines, surgery is a potential option for BPH patients with severe LUTS or other complications, including recurrent urinary tract infections, AUR, renal insufficiency, recurrent bladder stones, gross hematuria due to BPH, or those who are unwilling to receive drug treatments (Foster et al., 2019). Although the standard surgical treatment for BPH is still transurethral resection of the prostate (TURP), other less invasive surgical therapies are available for patients with severe LUTS, including transurethral incision of the prostate, transurethral laser prostatectomy, transurethral microwave therapy of the prostate, and prostatic urethral lift (Foster et al., 2019).

## Drugs for BPH

### α1 Adrenergic Antagonists (α1-Blockers)

#### Mechanisms Underlying the Action of α1-Adrenergic Antagonists in the Treatment of BPH

α1-adrenoceptors are highly expressed in the smooth muscle cells of the prostate gland, bladder neck, and urethra. When stimulated by α-adrenergic nerve fibers, these cells cause strong contractions, resulting in increased levels of urethral resistance (Akinaga and García-Sáinz, 2019). Based on this physiological mechanism, α1-adrenergic antagonists bind to α1-adrenoceptors on the smooth muscle cells of the urethra and cause relaxation in smooth muscle tone, thereby reducing urethral resistance and relieving LUTS (Andersson and Gratzke, 2007). The α1-adrenoceptors can be classified into three different subtypes (α1a, α1b, and α1d); these subtypes are distributed across various anatomical sites (Akinaga and García-Sáinz, 2019). Consequently, α1-blockers can cause a range of side effects when used for the treatment of BPH, including postural hypotension, dizziness, asthenia, abnormal ejaculation, and intraoperative floppy iris syndrome (IFIS) (Andersson and Gratzke, 2007). Several α1-blockers have been approved by the FDA for the treatment of BPH, including alfuzosin, doxazosin, silodosin, tamsulosin, and terazosin.

#### The Efficacy of α1-Blockers in the Treatment of BPH

α1-blockers have become the most common form of drug prescribed by urologists to treat BPH, and can lead to obvious improvements in patients with LUTS. Previous clinical trials have reported that α1-blockers reduced IPSS by 50%, and increased the maximum urinary flow rate (Q-max) by 40% (Michel et al., 1998; Djavan et al., 2004). α1-blockers can significantly improve the urinary symptoms of patients, including voiding symptoms and storage symptoms. Moreover, these effects can be achieved within only a few weeks. Several clinical trials have reported that α1-blockers can be efficacious over both the short and long terms, and in some cases, can exert effect in a rapid manner (Masumori et al., 2013; Manjunatha et al., 2016). These drugs target only α-adrenoceptors and do not induce changes in the size of the prostate, particularly within the transition zone (Roehrborn, 2006).

Interestingly, BPH patients who have a smaller prostate volume (PV) appear to represent optimal candidates for treatment with α1-blockers alone, while BPH patients with a larger PV are more suitable for treatment with 5α-reductase inhibitors (e.g., dutasteride) or combination therapy (e.g., dutasteride with tamsulosin). A previous prospective study, conducted in a single center, investigated the effects of silodosin monotherapy on LUTS, with specific reference to prostate size. In this previous study, 140 outpatients were equally divided into two groups: a small prostate group (SP, PV < 40 ml) and a large prostate group (LP, PV≥40 ml). Both groups were given silodosin (8 mg/day) for two years. The authors recorded IPSS, overactive bladder symptoms score (OABSS), bladder outlet obstruction index (BOOI), and detrusor overactivity at baseline, 3 months, and 24 months after treatment. Both groups showed significant improvements in IPSS and parameters related to the function of the lower urinary tract (storage function parameters and voiding function parameters), particularly in the SP group (PV < 40 ml). Although voiding function declined over time, the benefits in storage function were maintained for 2 years in the LP group. It is possible that the increase in bladder flow caused by α1-adrenoceptor antagonists led directly to an improvement in storage function, and that the greater structural changes taking place in the prostate stroma in BPH patients with a larger PV resulted in an improvement in BOOI (Matsukawa et al., 2018). Similarly, a randomized controlled intervention study reported that α1-blockers worked better in men with a PV ≤ 35 ml compared with those with larger PV (Joo et al., 2012).

In addition to patients with a small PV, α1-blockers appear to be more appropriate for BPH patients with lower baseline PSA levels; BPH patients with higher PSA levels appear to be more suitable for treatment with 5-ARIs or a combination of 5-ARIs and α1-blockers. In a parallel controlled trial, there was no remarkable improvement in terms of IPSS when compared between tamsulosin (an α1-blocker) and dutasteride (a type of 5-ARI) in patients with a baseline PSA level < 3.5 ng/ml; however, patients with baseline PSA levels > 3.5 ng/ml and treated with dutasteride showed significant improvement in IPSS compared to those treated with tamsulosin (Roehrborn et al., 2009).

#### Potential Side Effects Associated With the Use of α1-Blockers in BPH Treatment

In general, different α1-blockers appear to have similar effects on patients with LUTS; however, there is considerable variation in terms of side effects. Based on their binding affinity to α1-adrenoceptor subtypes, α1-blockers can be divided into selective α1-blockers and nonselective α1-blockers. The nonselective α1-blockers include terazosin, doxazosin, and alfuzosin; these drugs can block all α1-adrenoceptor subtypes equally. Selective α1-blockers include silodosin and tamsulosin. However, alfuzosin appears to be an exception. When we consider affinity to α1-adrenoceptor subtypes, alfuzosin can be referred to as a nonselective α1-blocker; however, this drug is clinically uroselective and has no significant effect on vascular α-adrenoceptors (Lowe, 2004).

α1-blockers are associated with three major side effects. Firstly, α1-blockers are associated with cardiovascular events such as postural hypotension, asthenia, and dizziness. The mechanism underlying these cardiovascular side effects might involve the reduction of blood pressure caused by α1-blocker-induced vasodilatation in smooth muscle cells and endothelial cells (Akinaga and García-Sáinz, 2019). However, De Mey and colleagues report that asthenia and dizziness reported in patients taking α1-blockers were not associated with blood pressure changes, but more likely to be associated with the central nervous system, which is also known to express α1-adrenoceptors (De Mey, 2000). Almost all α1-blockers, but particularly terazosin and doxazosin, can cause these side effects, although those related to selective α1-blockers are relatively weaker (Oelke et al., 2014a). Therefore, it is important to evaluate cardiovascular function in patients with BPH before considering the use of α1-blockers.

The second side effect is ejaculatory dysfunction (EjD). α1-blockers are known to cause retrograde ejaculation, but do not affect libido. Several meta-analyses have reported that tamsulosin and silodosin have the most significant association with retrograde ejaculation; other α1-blockers, such as alfuzosin, doxazosin, and terazosin, have a far smaller impact on ejaculatory function (Rosen et al., 2009; Gacci et al., 2014; Jung et al., 2017). In a previous randomized controlled trial, analysis of covariance (ANCOVA) was used to compare a group of patients taking silodosin with controls in order to analyze changes in Q-max, IPSS, and treatment efficacy. Over the short term, silodosin treatment led to a rapid improvement in urinary symptoms and a reduced risk of postural hypotension; however, the most common side effect related to silodosin treatment was retrograde ejaculation (Marks et al., 2013). Several other meta-analyses have shown that silodosin is stronger than tamsulosin when considering the improvement of urinary symptoms, particularly with regards to voiding symptoms; however, these studies also reported that silodosin was associated with a much higher risk of retrograde ejaculation (Wu et al., 2013; Gacci et al., 2014). The difference between these two α1-blockers with regards to their respective efficacy in patients with LUTS may be attributable to the significantly higher affinity between silodosin and α1a-adrenoceptors (Tatemichi et al., 2006). In general, silodosin had the strongest effects with regards to the side effect of retrograde ejaculation, followed by tamsulosin and nonselective α1-blockers. Interestingly, a very recent systematic review featuring data from 1,371 patients in six cohort studies provided evidence that alfuzosin could improve ejaculatory function (Yeung et al., 2020). It is possible that the blockade of adrenoceptors located in spermatic ducts and seminal vesicles may be associated with EjD in BPH patients receiving α1-blockers (Hisasue et al., 2006). The specific mechanisms underlying these observations may include insufficient contraction of the seminal vesicles, insufficient rhythmic contraction of the muscles of the pelvic floor, and the loss of seminal emission (Kobayashi et al., 2008; Nagai et al., 2008).

The third side effect that is commonly associated with α1-blockers is IFIS. This side effect often occurs in patients taking α-blockers who undergo cataract surgery; the risk of IFIS, however, is relatively low. It has been reported that most α1-blockers have the ability to cause IFIS, and that the highest level of risk is associated with tamsulosin (Srinivasan et al., 2007). IFIS also occurs in patients that have discontinued α-blockers (Nguyen et al., 2007). It is not yet known how α-blockers can induce IFIS. However, it is possible that the blockade of α-adrenoceptors could lead to contraction of the iris dilator muscle, or that the interaction between α-blockers and melanin induces atrophy in the dilator muscle (Oelke et al., 2014a). Therefore, prior to cataract surgery, it is important that ophthalmologists include previous α-blocker use in their patient history, and, if necessary, prohibit the use of α-blockers, especially tamsulosin, during cataract surgery.

α1-blockers have become the first-line clinical treatment for BPH and are commonly used worldwide. However, these drugs are mainly suitable for patients with moderate-to-severe LUTS, and are particularly useful for patients with a small PV and low baseline PSA levels. However, α1-blockers have a range of side effects; consequently, alfuzosin may be the most appropriate drug to prescribe to younger LUTS-BPH patients who are sexually active. This is because alfuzosin has been proven to improve ejaculatory function, while both silodosin and tamsulosin are contraindicated. Nonselective α1-blockers would not be appropriate for patients experiencing postural hypotension, asthenia, dizziness, and other risk factors associated with cardiovascular events. Patients scheduled to undergo cataract surgery should not be administered α-blockers, particularly tamsulosin.

### 5α-Reductase Inhibitors (5-ARIs)

#### Mechanisms Underlying the Action of 5-ARIs in the Treatment of BPH

It is commonly known that testosterone and dihydrotestosterone (DHT) promote the development of BPH. DHT plays a primary role in the development of BPH and amplify androgen-related activities in the prostate. 5α-reductase inhibitors (5-ARIs) were developed to treat BPH on the basis that testosterone can be converted into DHT by the enzyme 5α-reductase. The 5-ARIs can inhibit the conversion of testosterone and reduce the levels of DHT, thereby shrinking an enlarged prostate and preventing the progression of disease into BPH (Drobnis and Nangia, 2017). 5α-reductases can be divided into two subtypes, with very different patterns of biodistribution: type I and type II. The type I 5α-reductases are widely expressed in the skin, sebaceous glands, liver, and prostate epithelial cells. Type II 5α-reductases are mainly expressed in the stromal cells of the prostate, and, to a lesser extent, the epithelial cells (Cilotti et al., 2001). There are two inhibitors of 5α-reductase: finasteride and dutasteride. Finasteride is specific to type II 5α-reductase, while dutasteride inhibits both type I and type II 5α-reductase (Drobnis and Nangia, 2017).

#### The Efficacy of 5-ARIs in the Treatment of BPH

It is known that 5-ARIs can significantly reduce PV and serum levels of PSA. In a previous study, Na et al. investigated the safety and efficacy of dutasteride treatment in Chinese BPH patients using a double-blind, parallel placebo-controlled clinical trial. This study specifically recruited patients with a total prostate volume (TPV) of 30 ml or more; Q-max ranged from 5 ml/s to 15 ml/s; and the American Urology Association Symptom Index (AUA-SI) score was 12 units or higher. The research subjects were randomly classified into a dutasteride group or a placebo group. The dutasteride group was administered 0.5 mg of dutasteride on a daily basis, while the placebo group was given the placebo. After six months, the total PV had reduced by an average of 17.14% and 3.71% in the dutasteride group and the placebo group, respectively. Patients in the placebo group were given 0.5 mg of dutasteride on a daily basis for six months; the mean TPV had fallen by 11.75% when assessed at 12 months, and by 14.65% when assessed at 18 months. The dutasteride group also showed greater mean reductions in TPV at 12 months (18.83%) and 18 months (22.85%). This study provided strong evidence that 5-ARIs can reduce PV over the long term in men with BPH (Na et al., 2012).

Another clinical study examined the effect of 5-ARIs on serum PSA levels and PV. In this study, all 166 BPH patients were administered 5 mg of finasteride on a daily basis for six months. Researchers then measured the median percentage change in serum PSA levels in the entire study cohort, and measured PV by transrectal ultrasonography and prostate-specific antigen density (PSAD) in 86 men with BPH. In another study, Chiu and Yong reported that the median percentage changes in PSA level, PV, and PSAD, were −44.26%, −17.80%, and −38.67%, respectively (Chiu and Yong, 2004). Other studies have shown that 5-ARIs can also significantly improve Q-max, QOL, LUTS, and nocturia in men with BPH (Na et al., 2012; Oelke et al., 2014b).

Patients with enlarged prostates and higher baseline PSA levels are usually at greater risk of prostatic hyperplasia progression. In addition, LUTS deterioration, AUR, and BPH-related surgery are all closely associated with the clinical progression of BPH (Roehrborn, 2008). Several reports have confirmed that the long-term treatment of BPH patients with 5-ARIs, finasteride, and dutasteride can lead to reductions in the risk of developing AUR, and a reduction in the need for BPH-related surgery (Roehrborn and Ray, 2006; Kaplan et al., 2011). It is particularly evident that 5-ARIs can significantly prevent the progression of BPH.

The 5-ARIs are most suitable for patients with moderate-to-severe LUTS-BPH and an enlarged prostate. It is also evident that the baseline PV is closely associated with the outcome of BPH patients treated with 5-ARIs. In a four-year clinical trial, Roehrborn et al. compared the influence of baseline PV and PSA on the incidence of AUR, BPH-related surgery, and overall clinical progression in 4844 men treated with tamsulosin, dutasteride, or a combination of both drugs. Analysis showed that there was a greater reduction in the risk of AUR or BPH-related surgery in patients with a baseline PV ≥ 40 mL and receiving dutasteride than in patients with a smaller PV (Roehrborn et al., 2011). Kaplan et al. also reported that LUTS-BPH patients with a PV > 30 ml are ideal candidates for finasteride therapy (Kaplan et al., 2011). However, neither of these two clinical studies observed any significant improvement in terms of clinical progression in patients with a smaller PV. In addition, total serum PSA represents a significant predictor for evaluation of the efficacy of 5-ARIs in BPH patients. Several studies have reported that BPH patients with higher baseline PSA levels are more suitable for treatment involving 5-ARIs (Roehrborn et al., 2009; Roehrborn et al., 2011). These two previous studies showed that remarkable therapeutic effects can be achieved in men who receive 5-ARIs over long periods of time. Interestingly, 5-ARIs have been shown to cure androgenetic alopecia; dutasteride has been shown to be more effective than finasteride in the treatment of androgenetic alopecia (Gubelin Harcha et al., 2014).

#### Potential Side Effects Associated With the Use of 5-ARIs in the Treatment of BPH

5-ARI treatments are associated with a range of side effects in BPH patients, including decreased libido, gynecomastia, and erectile dysfunction (ED). There are also reports that dutasteride can improve the prognosis of patients with prostate cancer, especially in low-risk prostate cancer (Fleshner et al., 2012; Schröder et al., 2013). Similarly, the Prostate Cancer Prevention Trial (PCPT) provided evidence that finasteride had similar effects to dutasteride in terms of reducing the incidence of prostate cancer, although finasteride can also increase the risk of high-grade Gleason prostate tumors (Thompson et al., 2003). For this reason, it appears that the genetic instability created by alteration in the androgen milieu results in an invasive prostate cancer phenotype (Tso et al., 2000). Generally, there are no obvious differences between dutasteride and finasteride in terms of efficacy and safety, although finasteride has fewer sexual side effects and breast complications than dutasteride when used in the treatment for BPH (Kaplan et al., 2012).

### Muscarinic Receptor Antagonists (MRAs)

#### Mechanisms Underlying the Use of MRAs in the Treatment of BPH

Abundant M2 and M3 cholinergic receptors are distributed on the smooth muscle cells of the bladder, urothelium, and afferent nerves. The detrusor contracts when these cholinergic receptors are stimulated, resulting in frequent micturition and an increased urgency to urinate (Eglen, 2005). Although the M2 receptor subtype is far more abundant in the bladder than the M3 receptor subtype, the M3 subtype is more crucial to bladder contractions than the M2 subtype (Chapple et al., 2002). It is clear that M2/M3 cholinergic receptors play significant roles in the urethra and bladder. Consequently, MRAs were developed to ameliorate storage symptoms in BPH patients. MRAs exert action by blocking muscarinic cholinergic receptors, although the exact mechanisms and sites of action have yet to be established (Sellers and Chess-Williams, 2012). Thus far, several MRAs have been approved by the FDA, including oxybutynin, tolterodine, fesoterodine, darifenacin, solifenacin, and trospium. This class of drugs is primarily used to treat the storage symptoms associated with LUTS and overactive bladder (OAB), such as frequent micturition, urgency of urination, and urgent urinary incontinence (UUI) (Yamada et al., 2018).

#### The Efficacy of MRAs in the Treatment of BPH

The most significant strength of MRAs is their ability to improve storage in patients with LUTS. Lee et al. described a prospective, randomized, multicenter, study in which 156 patients (IPSS ≥14, voiding subscore ≥ 8, storage subscore ≥ 6) were randomized into two groups. In group 1 (n=80), the patients were received 0.2 mg of tamsulosin each day for four weeks, and then received 0.2 mg of tamsulosin plus 5 mg of solifenacin on a daily basis for 8 weeks. In group 2, all patients received a combination of 0.2 mg of tamsulosin and 5 mg of solifenacin on a daily basis for 12 weeks. The authors assessed changes from baseline in a range of parameters, including total IPSS, IPSS storage sub-score, IPSS voiding sub-score, OABSS, and urgency symptoms between the two groups over the twelve weeks of treatment. Analysis showed that the IPSS storage sub-score was obviously lower in the group of patients receiving a daily combination of 0.2 mg tamsulosin plus 5 mg solifenacin than in the group receiving 0.2 mg of tamsulosin each day over a period of 4 weeks. However, there were no striking differences with regards to total IPSS or IPSS voiding sub-score between the two groups at four weeks. After 12 weeks of treatment, there were no significant differences between the two groups with regards to storage symptoms (Lee et al., 2014). In another meta-analysis, Gong et al. also provided strong evidence to support the efficacy of a combination therapy featuring MRAs and α1-blockers when used for patients with LUTS (Gong et al., 2015).

Interestingly, it appears that LUTS patients with a smaller PV or a lower baseline PSA level would achieve better benefits from antimuscarinic therapy than those with a larger PV or higher PSA level (Roehrborn et al., 2008; Liao and Kuo, 2015). Liao and Kuo found that, among in patients with a TPV > 40 ml, there were no significant differences between patients treated with tolterodine and those treated with doxazosin with regards to IPSS improvement (76.5% *vs.* 78.0%, p = 0.871). In contrast, there was a significant improvement in IPSS in the group treated with tolterodine compared with that in the group treated with doxazosin (73.3% *vs.* 57.6%, p = 0.040) among patients with a TPV < 40 ml (Liao and Kuo, 2015). In addition, a randomized, double-blind, research study reported that the efficacy of antimuscarinic drugs in BPH patients was closely related to the serum PSA level. Roehrborn et al. found that tolterodine could significantly improve 24-h frequency, frequency-urgency sum, and IPSS storage sub-score in patients with serum PSA levels < 1.3 ng/ml in comparison with those receiving placebo (Roehrborn et al., 2008).

The specific formulations of anti-muscarinic drugs are also associated with their relative efficacies. For example, tolterodine is available in two drug formulations: extended-release (ER) and immediate-release (IR). The ER formulation of tolterodine is more effective than the IR formulation when used for the treatment of OAB symptoms, and is also associated with a lower incidence of side effects (Van Kerrebroeck et al., 2001).

#### Side Effects of MRAs in the Treatment of BPH

There are several side effects associated with MRAs, including dry mouth, pruritus, constipation, micturition difficulties, nasopharyngitis, and dizziness. A previous meta-analysis showed that the most common side effect is dry mouth (Chapple et al., 2008). An open trial showed that oxybutynin is more likely to cause dry mouth than tolterodine, and that although there were similarities between the two agents in terms of other side effects, oxybutynin is more effective than tolterodine for reducing micturition (Diokno et al., 2003). Interestingly, a clinical study found that the combination of tolterodine with pilocarpine led to an effective reduction in the risk of dry mouth and did not simultaneously influence the efficacy of tolterodine in the treatment of OAB (Dmochowski et al., 2014).

However, the most serious side effect of antimuscarinic drugs is AUR. Because of their effect on the bladder (detrusor flaccidity), antimuscarinics are closely associated with AUR. A retrospective cohort study suggested that patients receiving antimuscarinic treatment for one month had a higher incidence of AUR than patients receiving long-term treatment (8.3 *vs.* 2.0) (Martín-Merino et al., 2009). The risk of AUR also seems to be higher during the early stages of antimuscarinic treatment. In terms of the risk of AUR, another meta-analysis, involving randomized controlled trials (RCTs) and observation studies, 365 men, and a 12-week follow-up period, suggested that the incidence of AUR was only small (0.3%) (Blake-James et al., 2007). According to the AUA Guidelines on the Management of Benign Prostatic Hyperplasia, LUTS-BPH patients with a PVR > 250 to 300 ml should only receive antimuscarinic treatment with caution. The AUA guideline also recommends that MRAs should only be prescribed after investigating a patient's PVR (Mcvary et al., 2011). Another meta-analysis investigated the relationship between age and the safety of antimuscarinics in older adults. It appears that the treatment of LUTS patients with antimuscarinics increases the risk of side effects in patients aged 65 or older when compared with patients younger than 65 years of age (Vouri et al., 2017). This result may be associated with drug metabolism and the fact that elderly individuals generally exhibit lower rates of metabolism, thus prolonging the drug residence time.

Several newer antimuscarinics have been developed for use in LUTS-BPH patients; these include tarafenacin, imidafenacin, vaginal oxybutynin, and tolenix (a combination therapy involving 2 mg of tolterodine IR and 9g of ER pilocarpine 9 mg). Although these drugs have been demonstrated to reduce the side effects of traditional MRAs in clinical trials, including dry mouth and constipation, their ability to relieve storage symptoms remains invariant, or slightly better, in LUTS-BPH patients (Thiagamoorthy et al., 2016). However, these drugs are still not approved by the FDA for BPH treatment, although imidafenacin has been licensed in Japan.

### β3-Adrenoceptor Agonists (Beta-3 Agonists)

#### Mechanisms Underlying the Use of β3-Adrenoceptor Agonists in BPH Treatment

β-adrenoceptors are ubiquitously expressed in the bladder, urethra, and prostate. Furthermore, the expression levels of the β3-adrenoceptor subtype are far higher than those of the other two subtypes (β1-adrenoceptor and β2-adrenoceptor) in the bladder (Michel and Vrydag, 2006). β3-adrenoceptor agonists were first developed to reduce detrusor tone and promote urine storage on the basis that activation of the β-adrenoceptors relaxes the smooth muscle and detrusor in both the urethra and bladder (Yang and Tao, 2019). Mirabegron is currently the only selective β3-adrenoceptor agonist that has been approved by the FDA.

#### The Efficacy of β3-Adrenoceptor Agonists in BPH Patients

Mirabegron has been shown to significantly improve OAB symptom-frequency, urgency, nocturia, and UUI-in patients with LUTS or/and OAB, but has little effect on voiding urodynamics, particularly maximum urinary flow and detrusor pressure at maximum urinary flow, compared with a placebo, over a period of 12 weeks (Nitti et al., 2013). Mirabegron is commonly used for the treatment of OAB syndrome induced by BPH owing to its pharmacological activity, although currently AUA does not recommend its use for BPH treatment. This drug is often used as a second-line medicine for LUTS treatment. Because of the similarity between β3-adrenoceptor agonists and muscarinic antagonists in terms of pharmacological action, mirabegron is regarded as a well-tolerated alternative to MRAs for the treatment of LUTS (Tubaro et al., 2017).

#### The Side Effects of β3-Adrenoceptor Agonists in BPH Treatment

The most common side effects induced by mirabegron treatment include headache, constipation, nasopharyngitis, hypertension, and urinary tract infection, with the latter three being the most common among these (Chapple et al., 2014). A previous meta-analysis investigated the tolerability of mirabegron in comparison with antimuscarinics and combination therapy, and suggested that 50 mg of mirabegron had a lower risk of side effects than antimuscarinic monotherapy, but with similar efficacy (Kelleher et al., 2018). Aside from having fewer side effects than antimuscarinics, mirabegron is also associated with greater levels of persistence than antimuscarinics. A retrospective and observational study provided evidence that patients with OAB showed greater levels of persistence and adherence when treated with mirabegron than those treated with tolterodine (Chapple et al., 2017). It is also worth noting that uncontrolled hypertension is a contraindication due to the risk of severe hypertension (Michel and Gravas, 2016). Therefore, it is important that urologists review the cardiovascular disease history of their patients and monitor blood pressure when prescribing mirabegron for the treatment of OAB.

### Plant Extracts

Plant extracts are a class of herbal medicines that remain somewhat controversial and are not yet recommended by the EAU or AUA for the treatment of BPH. Such treatments predominantly include extracts from *Saw palmetto*, *Cucurbita pepo* L., *Prunus africana*, *Urtica dioica* L, and *Secale cereale* L. (Sharma et al., 2017). Phytotherapies have not yet been accepted by the EAU and AUA because of methodological problems and heterogeneity in the clinical trials carried out thus far. However, plant extracts have already been prescribed in clinical practice for BPH patients who were unwilling to accept standard medical treatments in the United States and numerous European countries, including Hungary, Germany, Poland, and France (Cornu et al., 2010). The prescription of plant extracts is therefore dependent on the guidelines adopted by individual nations and their health systems.

Extracts from *S. palmetto* and *C. pepo* L. are the most frequently used. Over the past few decades, increasing clinical evidence has demonstrated the effects of these extracts in patients with BPH. One meta-analysis, which included data extracted from 15 RCTs and 12 observational studies, suggested that Permixon, a hexanic extract of *S. palmetto*, had similar levels of efficacy to α1-blockers with regards to improvements in the IPSS, QOL, and Q-max (Vela-Navarrete et al., 2018). This previous study also showed that Permixon treatment led to an improvement in LUTS symptoms, and an inhibition in the progression of BPH, that was equivalent to that achieved with a 6-month treatment with 5-ARIs (Vela-Navarrete et al., 2018). A 15-year open observation study investigated the changes in PV, Q-max, QOL, and IPSS, in BPH patients treated with a daily dose of *S. palmetto* extract (320 mg), and found that this extract was able to prevent the progression of BPH (Vinarov et al., 2019).

The *C. pepo* L. extract has also been reported to be efficacious for the treatment of BPH patients. In a pilot study, Leibbrand et al. assigned 60 BPH patients with an IPSS of 14.8 (95% confidence interval [CI]: 13.5–16.1) to a group that received an oil-free hydroethanolic *C. pepo* L. extract once daily for 12 weeks, and investigated the consequent changes in IPSS, frequency of nocturia, and PVR. Analysis showed that this treatment led to an average reduction of 30.1% (95% CI: 23.1–37.1) for total IPSS. There was also an obvious reduction in PVR (baseline: 83.67 mL [95% CI: 58.02–109.3]; after 12 weeks: 63.11 mL [95% CI: 45.37–80.85]), and a significant reduction in the frequency of nocturia (Leibbrand et al., 2019). Although the precise mechanisms underlying the effects of plant extracts on BPH patients have yet to be elucidated, several studies suggest that plant extracts may exert pharmacological action through anti-androgenic, anti-proliferative, anti-inflammatory, and anti-edema activities (Buck, 2004; Gandaglia et al., 2017; Paterniti et al., 2018).However, the mechanisms underlying these effects may involve the action of multiple active ingredients present in the plant extract.

The existing literature clearly suggests that plant extracts are effective for the treatment of BPH. However, some clinical trials have reported contradictory results. A double-blind trial investigating the efficacy of *S. palmetto* in 225 BPH patients with moderate-to-serve LUTS found no obvious difference between the control group and the treated patients (Bent et al., 2006). More recently, Russo et al. used surface under the cumulative ranking curve (SUCRA) analysis to compare the relative effects of alfuzosin, tamsulosin, silodosin, *S. palmetto*, and placebo, with regards to improvements in LUTS and peak flow. This research found that the score for *S. palmetto* in terms of the improvement of LUTS and peak flow in BPH patients was the lowest, and even lower than that of the placebo group. These results indicated that *S. palmetto* had no clinical effect in terms of improving LUTS and peak flow in BPH patients (Russo et al., 2020). To date, however, no other studies have indicated that *C. pepo* L. has no clinical effect as a form of treatment for BPH.

It is possible that the different clinical outcomes evident in the literature for the same plant extract may be related to the differences in brands from which the extracts were prepared, methods of extraction, environments in which the plants were grown, or time at which they were harvested. It is also possible that different parts or concentrations, of the plants were used to extract the active compounds (Habib and Wyllie, 2004). It is also worth noting that several studies have suggested that *S. palmetto* appears to have few negative impacts on sexual function; similar levels of activity have also been recorded for *Crocus sativus* L (Cai et al., 2013; Vela-Navarrete et al., 2018). At the time of writing this article, plant extracts have still not been approved by the FDA, EAU, or the AUA because of the associated drug heterogeneity, a limited regulatory framework, and the methodological limitations of the trials and meta-analyses published thus far (Mcvary et al., 2011).

### Phosphodiesterase 5 Inhibitors (PDE5Is)

#### Mechanisms Underlying the Use of PDE5Is in BPH Treatment

Phosphodiesterase 5 is distributed throughout the urinary tract, including the bladder detrusor, vascular smooth muscle, prostate tissue, and ureter. PDE5Is relax the urinary smooth muscle and the bladder detrusor, and can therefore improve LUTS by increasing the concentration of intracellular cyclic guanosine monophosphate (cGMP) (Fibbi et al., 2010). It is also worth noting that the nitric oxide (NO) pathway is the underlying signaling mechanism by which PDE5Is exert their pharmacological action on urinary smooth muscle (Kedia et al., 2008).

#### The Efficacy of PDE5Is in BPH Patients

Although several PDE5Is (sildenafil, tadalafil, and vardenafil) have been reported to exert efficacy on LUTS-BPH patients, only tadalafil has been approved by the FDA to treat patients with BPH (Hiramatsu et al., 2020). PDE5Is were initially developed to treat ED. Many reports have suggested that tadalafil is a highly effective option for the treatment of LUTS related to BPH. A previous meta-analysis, featuring 3214 patients with BPH, demonstrated that PDE5Is can significantly improve IPSS and International Index of Erectile Function (IIEF) scores, but have no effect on Q-max (Gacci et al., 2012). In a clinical trial, Porst et al. also demonstrated that tadalafil treatment resulted in significant improvements to the IPSS and IIEF scores, but had no impact on Q-max in LUTS-BPH patients (Porst et al., 2011). Therefore, tadalafil appears to be a good option for the treatment of sexually active LUTS-BPH patients with or without ED.

#### Side Effects of PDE5Is in BPH Treatment

The use of PDE5Is is associated with numerous side effects, including flushing, indigestion, headache, back pain, gastroesophageal reflux, and nasal congestion (Gacci et al., 2012). A previous meta-analysis revealed that headache, dyspepsia, and backpain were the most common side effect associated with PDE5Is, and that there were no significant differences between various types of PDE5Is in this respect (Yuan et al., 2013). Yuan et al. also found that the form of PDE5I with the highest risk of headache was avanafil; the PDE5I with the highest risk of indigestion was tadalafil; and the PDE5I with the highest risk of backpain was vardenafil; while tadalafil was associated with a higher risk of myalgia than sildenafil. (Yuan et al., 2013). Another meta-analysis assessed the effect of a daily 15 mg dose of tadalafil for the treatment of BPH and ED, and suggested that a 5-mg tadalafil, given once daily, was well tolerated due to the low risk of discontinuation resulting from related side effects (Wang et al., 2018). The side effects associated with PDE5Is appear to be moderate and well tolerated by BPH patients, although contraindications should be considered by urologists. When combined with nitrates, PDE5Is can increase the risk of life-threatening symptomatic hypotension (Chrysant, 2013); therefore, it is important that nitrates should not be used in combination therapies with PDE5Is. According to a clinical study, the interval of time between the intake of nitrates and the intake of long-acting PDE5I tadalafil should be at least 48 h (Abrams, 2004). The α1-blockers, doxazosin and terazosin, can have similar effects on patients taking PDE5Is. In addition to the drugs described above, diuretics can also exert influence on the efficacy of tadalafil. Among the population of patients with BPH, there is a sub-population who are elderly and have hypertension. A previous integrated analysis of data from four RCTs investigated the influence of cardiovascular risk factors and antihypertension drugs on a daily dose of oral tadalafil (5 mg) in patients with BPH. Among the patients taking antihypertensive drugs on a daily basis, the placebo-adjusted least squares (LS) mean improvement in total IPSS in patients taking diuretics was −0.2 (95% CI: −2.1 to 1.7), the placebo-adjusted LS mean improvement in total IPSS in patients taking other forms of antihypertension medicine was −2.8 (95% CI, −3.7 to −1.9), and the placebo-adjusted LS mean improvement in total IPSS in patients not taking any antihypertension medicine was −2.3 (95% CI, −3.2 to −1.5). These data indicate that diuretics have a negative influence on the efficacy of tadalafil treatment in LUTS-BPH patients who take daily doses of antihypertensive medication (Vlachopoulos et al., 2015). Serious cardiovascular events are another problematic contraindication that should be considered carefully when prescribing this drug (Chrysant, 2013). Urologists should evaluate the general health of BPH patients before prescribing tadalafil, particularly with regards to cardiovascular disease.

## Combination Therapies for BPH

In LUTS-BPH patients, it is often difficult to achieve satisfactory efficacy with a single drug, and patients often discontinue treatment because of side effects. Consequently, there is an urgent need to develop novel combination therapies with satisfactory efficacy and inhibition of disease progression as well as improved patient adherence to treatment. The combination of different drugs may result in additional side effects, however. Currently, the most common combination therapies include the combination of α1-blockers with 5-ARIs and with MRAs. The most commonly used combination therapy is α1-blockers combined with 5-ARIs; furthermore, this is the only combination therapy recommended by the AUA (Mcvary et al., 2011). A previous clinical trial investigated the efficacy and safety of tamsulosin, dutasteride, and combination therapy, in the long-term treatment of patients with BPH over a four-year period. Results from this study showed that the combination of tamsulosin-dutasteride significantly reduced LUTS, delayed the disease progression of BPH, and reduced the risk of developing AUR and the need for surgery compared with a single-drug therapy; however, the incidence of side effects increased for the combination therapy (Roehrborn et al., 2010). According to AUA recommendations, the combination of an α1-blocker and a 5-ARI is more appropriate for patients with cases of LUTS that are associated with demonstrable prostatic enlargement (Mcvary et al., 2011). In addition, another meta-analysis, featuring data from 3,063 patients and seven articles, showed that treatment with a combination of tamsulosin and solifenacin achieved good efficacy in male LUTS-BPH patients, especially those with severe symptoms relating to urinary storage. However, attention should be paid to the PVR of patients when considering combination therapies in order to avoid increasing the risk of AUR (Gong et al., 2015). In addition to these common combination treatments, the combination of tadalafil with tamsulosin can also improve the IPSS, QOL, IIEF score, Q-max, and reduce the PVR more effectively than tamsulosin or tadalafil alone; however, this combination therapy also increases the risk of severe hypotension (Singh et al., 2014). The combination of PDE5Is with 5-ARIs may additionally represent a promising form of therapy (Roehrborn et al., 2015).

## Conclusion

Herein, we summarize the most commonly used drugs used to treat BPH. The most common forms of clinical treatment include α1-blockers, 5-ARIs, and the combination of these two drug types. The mechanism of action for each type of drug, along with its specific target, can vary widely. Furthermore, the drugs used to treat BPH exhibit a wide range of clinical applications, pharmacological actions, and side effects. It is also possible that the efficacy and side effects of each BPH drug may vary within the same patient population, and that the sensitivity of different BPH patient populations to the same BPH drug may also vary. Consequently, there is no universal therapy for LUTS-BPH patients, and individualized treatment plans are required to ensure the judicious use of medical resources. Before making decisions to choose the best available treatment for BPH patients, urologists should consider the severity of storage or voiding symptoms, the risk of disease progression (PV, baseline serum PSA level), patient characteristics (age, health condition, concomitant disease, current medication status, and the need for sexual function), individual preferences, and the acceptance of potential side effects.

## Author Contributions

**Figure 1:**
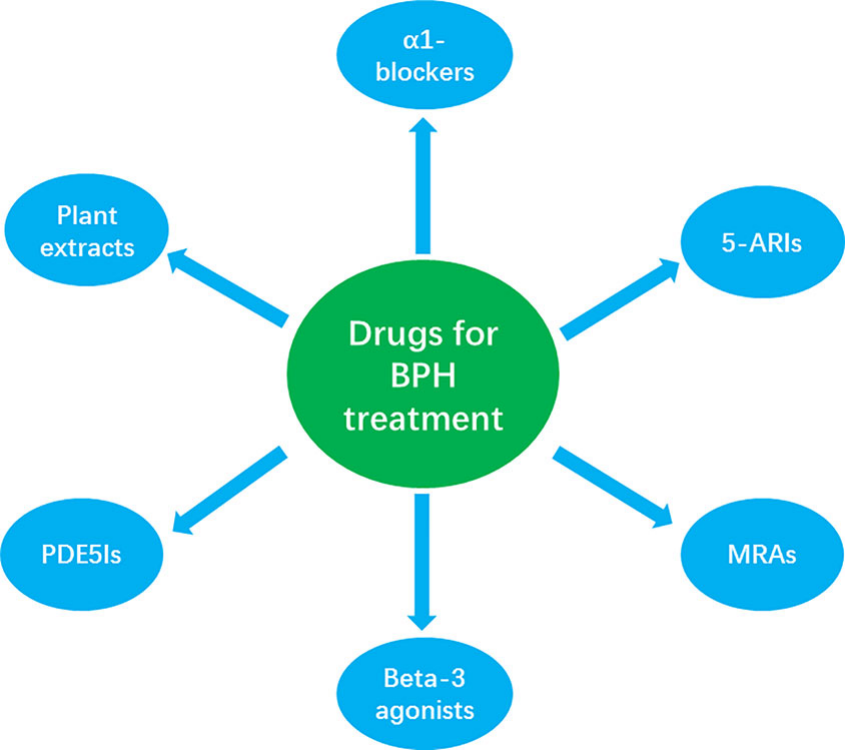
The classification of the most common drugs used for the clinical treatment of LUTS/BPH, showing α1-blockers (α1 adrenergic antagonists), 5α-reductase inhibitors (5-ARIs), muscarinic receptor antagonists (MRAS), β3-adrenoceptor agonists (beta-3 agonists), and phosphodiesterase 5 inhibitors (PDE5Is).

Z-JY reviewed the literature and wrote the first draft of the manuscript. **Figure 1** and **Table 1** were created by F-HX, H-CC, and L-HD. H-LY checked and revised the draft manuscript. All authors contributed read, revised, and approved the submitted version.

**Table 1:** The efficacy and safety of several common drugs approved by the FDA for the treatment of LUTS/BPH.

Drug	Type	Mechanism	Dosage forms	Efficacy	Adverse reactions (TOP 3)	Adverse reactions (general)	Recommendation and Strenght rating	Contraindication	Preacaution (specific)	Precaution (general)
Tamsulosin	Selective α1-blockers	Relaxes smooth muscle in prostate and bladder neck and decreases urethral resistance through the blockade of α1-adrenergic receptors	Tamsulosin 0.4mg	Treatment of BPH a. Improving LUTS, Q-max and QOL rapidly b. Maintaining long-term efficacy c. Terazosin and doxazosin are also anti-hypertensive drugs.	a.Headache b.Dizziness c.Abnormal Ejaculation	a.Cardiovarscular events Dizziness 1.the most common adverse reaction of α1-blockers 2.the most common: terazosin, doxazosin Postural hypotension most common agents: terazosin, doxazosin less common agents: alfuzosin, tamsulosin least common agents: silodosin Asthenia b.EjD most common agents: silodosin, tamsulosin less common agents: terazosin, doxazosin least common agents: alfuzosin c.IFIS most common agents: tamsulosin others are relatively low. d.Nonselective α1-blockers have more side effects than selective α1-blockers.	a.Offer α1-blockers to men with moderate-to-severe LUTS (Strength rating: Strong)	Patients known to be hypersensitive to tamsulosin.	a.Orthostasis b.Drug-Drug Interactions: strong inhibitors of CYP3A4 (ketoconazole), CYP2D6 inhibitors (paroxetine,terbinafine), cimetidine, other α-blockers, warfarin.	a. α1-blockers do not prevent the occurrence of urinary retention or need for surgery (all five α1-blockers) b.PDE5Is: The combination of α1-blocker and PDE5Is may cause symptomatic hypotension (all five α1-blockers) c. IFIS:Ophthalmologists should be informed about α1-blocker use prior to cataract surgery (all five α1-blockers) d. EjD: Sexually active patients treated with selective α1-blockers should be counselled about the risk of EjD (silodosin and tamsulosin) e.Dose titration and blood pressure monitoring. (doxazosin and terazosin) f.Priapism:All α1-blockers may cause priapism, although the risk is low.
Silodosin			Silodosin 8mg		a.Retrograde Ejaculation b.Dizziness c.Diarrhea			a.Severe renal impairment (CCr < 30 mL/min) b.Severe hepatic impairment (Child-Pugh score ≥ 10) c. Concomitant administration with strong Cytochrome P450 3A4 (CYP3A4) inhibitors (e.g., ketoconazole, clarithromycin, itraconazole, ritonavir)	a.Postural hypotension may develop when beginning silodosin treatment. b. Hepatic or renal impairment c.Drug-Drug Interactions: CYP3A4 inhibitors	
Alfuzosin	Non-selective α1-blockers		Alfuzosin 2.5mg		a.Dizziness b.Upper respiratory tract infection c.Headache			a.Moderate or severe hepatic insufficiency b.CYP3A4 inhibitors: ketoconazole, itraconazole, and ritonavir c.Patients known to be hypersensitive to alfuzosin	a. Drug-Drug Interactions: NOT to be used in combination with other α-blockers. b.Coronary Insufficiency: Alfuzosin should be discontinued if the symptoms of angina pectoris newly appear or worsen. c. QT prolongation history	
			Alfuzosin 10mg							
Terazosin			Terazosin 1mg Dose titration		a.Dizziness b.Asthenia c.Postural hypotension			Patients known to be hypersensitive to terazosin	a.Syncope and ‘‘First-dose'' Effect: Treatment should always be initiated with a 1 mg dose, given at bedtime, then dosage should be increased slowly. b.Orthostatic Hypotension c.Anti-hypertensive drugs: significant hypotension should beconsidered when terazosin is administered concomitantly with other antihypertensive agents, especially the calcium channel blocker verapamil.	
Doxazosin			Doxazosin 1mg Dose titration		a.Dizziness b.Fatigue c.Hypotension			Patients known to be hypersensitive to doxazosin	a.Postural hypotension may develop when beginning doxazosin treatment. b. Drug-Drug Interactions: CYP3A4 inhibitors	
Dutasterde	5-ARIs (type I and type II 5-ARIs)	Inhibits the conversion of testosterone to DHT through the inbibition of type I and type II 5α-reductase	Dutasteride: 0.5mg	Treatment of BPH a. Reducing PV and the serum PSA level b. Improving Q-max, QOL, LUTS and nocturia c. Preventing disease progression d.Reduction in the risk of AUR and the need for surgical intervention.	a.Impotence b.Decreased libido c.Ejaculation dysfunction	a.No Significant difference between dutasteride and finasteride b.Finasteride has fewer sexual side effects and breast complications .	a.Use 5-ARIs in men who have moderate-to-severe LUTS and an increased risk of disease progression (e.g. prostate volume > 40 mL). (Strength rating: Strong) b.Counsel patients with regards to the onset of action (three to six months) of 5-ARIs. (Strength rating: Strong)	a.Women and children: contraindicated for use in women and children. b.Patients with known hypersensitivity to dutasteride, other 5-ARIs	a. Large PVR and/or severely diminished urinary flow may not be good candidates for 5-ARIs therapy b. Men being treated with dutasteride should not donate blood until at least 6 months have passed following their last dose. c. Caution should be used in the administration of dutasteride to patients with liver disease d. Care should be taken when administering dutasteride to patients taking potent, chronic CYP3A4 enzyme inhibitors(e.g., ritonavir)	a.5-ARIs are suitable only for long-term treatment (years), because of the slow onset of action. b.The effect of 5-ARIs on the serum PSA concentration needs to be considered in relation to prostate carcinoma screening and PSA measure. c.Exposure of Women-Risk to Male Fetus: women who are pregnant or may be pregnant should not handle. d.5-ARIs are not indicated for use in women and children.
Finasteride	5-ARIs (type II 5-ARIs)	Inhibits the conversion of testosterone to DHT through the inbibition of type II 5α-reductase	Finasteride 5mg		a.Impotence b.Decreased Libido c.Breast Enlargement			a.Women and children: contraindicated for use in women and children. b.Patients with known hypersensitivity to finasteride, other 5-ARIs	a.Increased risk of high-grade prostate cancer (Gleason score 8-10 prostate cancer) b.Mild effect on semen characteristics (not clinically meaningful)	
Tolterodine	MRAs	Relaxes smooth muscle in bladder through inhibition of muscarinic receptors	Tolterodine 1mg	a.Improving storage LUTS and nocturia b.Treatment of OAB	a.Dry mouth b.Constipation c. Headache	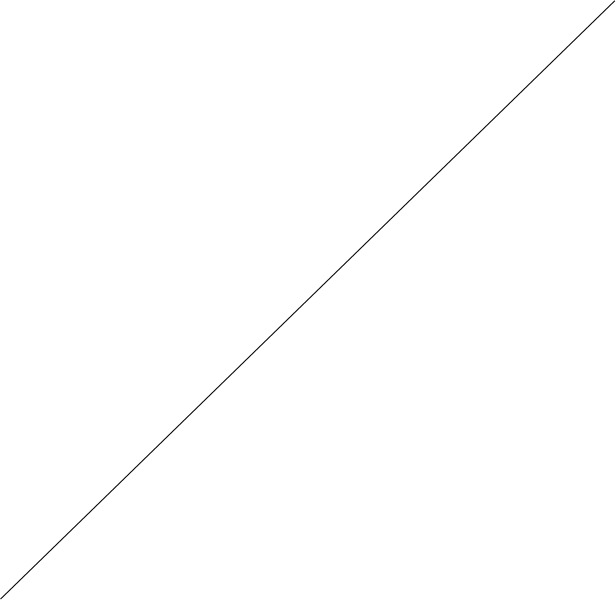	a. Use MRAs in men with moderate-to-severe LUTS who mainly have bladder storage symptoms. (Strength rating: Strong) b. Do not use MRAs in men with a PVR > 150 mL. (Strength rating: Weak)	a.Patients with urinary retention, gastric retention, or uncontrolled narrow-angle glaucoma. b.Patients with known hypersensitivity to tolterodine.	a.High risk of Urinary Retention: For patients with clinically significant bladder outflow obstruction. b.High risk of Gastric Retention:For patients with gastrointestinal obstructive disorders, such as pyloric stenosis. c.Decreased gastrointestinal motility: Patients with decreased gastrointestinal motility should be carefully treated by tolterodine d.Patients being treated for narrow-angle glaucoma e.CNS Effects: Patients should be monitored for signs of anticholinergic CNS effects, particularly after beginning treatment or increasing the dose. f.Patients with Myasthenia Gravis g.Patients with Congenital or Acquired QT Prolongation h.Anaphylaxis and angioedema may occour after the first dose of tolterodine	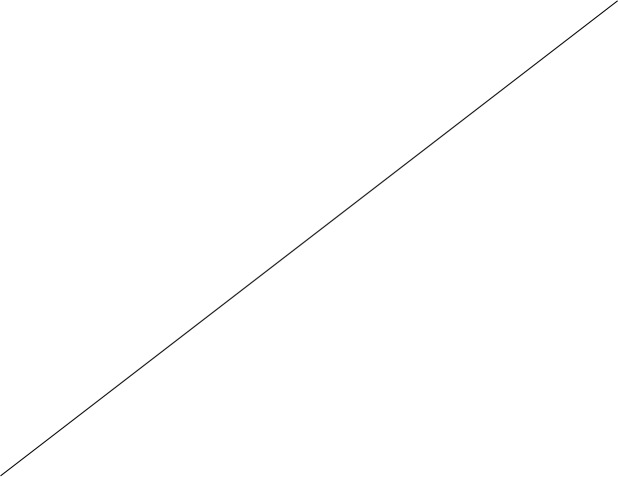
Mirabegron	Beta-3 agonists	Relaxes the detrusor smooth muscle during the storage phase of the urinary bladder fill-void cycle by activation of β3-adrenergic receptor	Mirabegron 25mg	a.Improving OAB b. Increasesing bladder capacity	a.Hypertension b.Nasopharyngitis c.Urinary tract infection	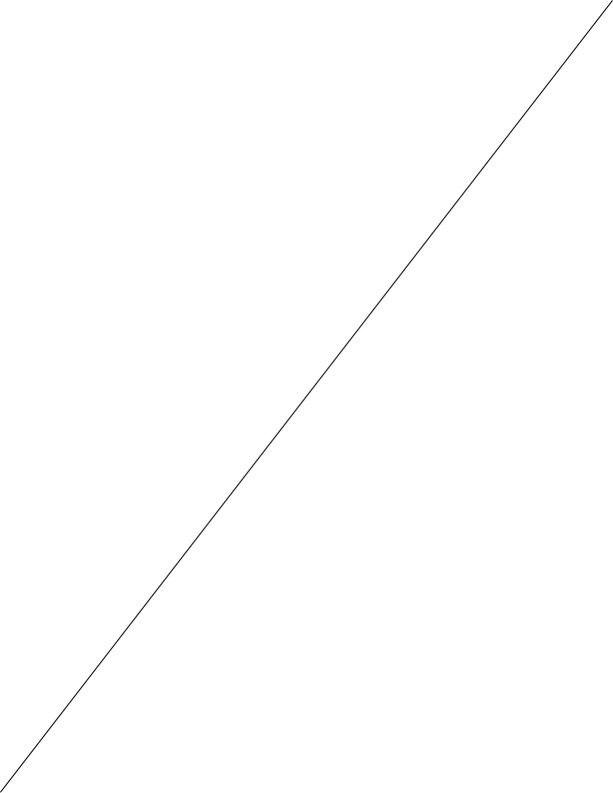	Use beta-3 agonists in men with moderate-to-severe LUTS who have mainly bladder storage symptoms. (Strength rating: Weak)	Patients with known hypersensitivity to mirabegron.	a.Patients with hypertension: Periodic determination of blood pressure is recommended, especially in hypertensive patients. b.Urinary retention may occur in patients with Bladder Outlet Obstruction c.Urinary retention may occur in patients taking muscarinic antagonist medications for the treatment of OAB d.Angioedema: Angioedema may occur after the first dose of mirabegron e.Drugs Metabolized by CYP2D6: Appropriate monitoring and dose adjustment may be necessary, with drugs metabolized by CYP2D6, such as thioridazine, flecainide, and propafenone.	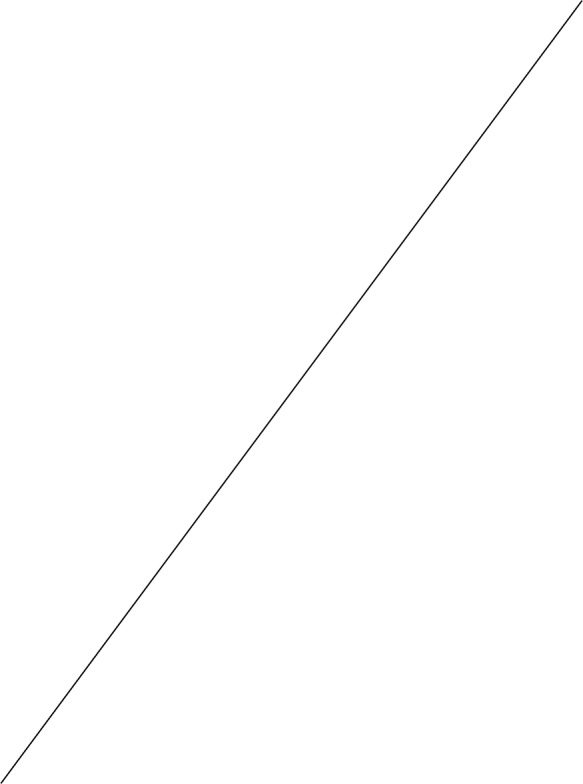
Tadalafil-Efficacy	PDE5Is	Relaxes urinary smooth muscle and the bladder detrusor by increasing the concentration of intracellular cGMP	Tadalafil 5mg	a. Treatment of BPH b. Treatment of ED c. Treatment of BPH and ED d. Combination therapy with finasteride for BPH e. Treatment of PAH	a.Headache b.Dyspepsia c.Back pain	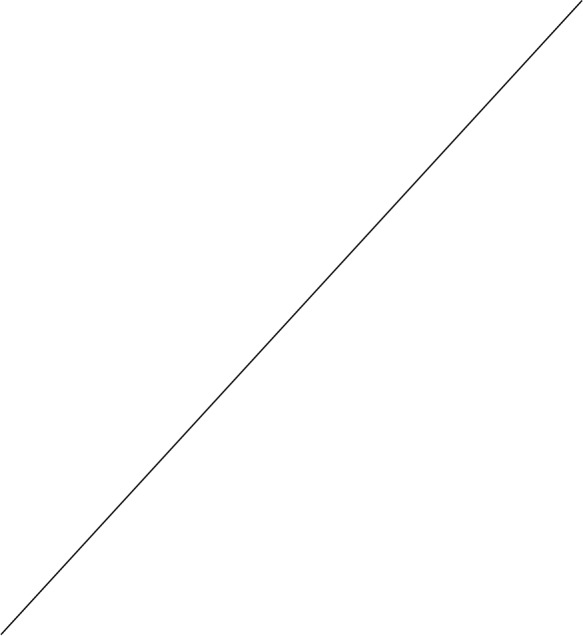	Use PDE5Is in men with moderate-to-severe LUTS with or without ED. (Strength rating : Strong)	a.Nitrates b.Patients with a known serious hypersensitivity to tadalafil c.Concomitant Guanylate Cyclase Stimulators, such as riociguat d.Cardiovascular disease : • myocardial infarction within the last 90 days • unstable angina or angina occurring during sexual intercourse • New York Heart Association Class 2 or greater heart failure in the last 6 months • uncontrolled arrhythmias, hypotension (<90/50 mm Hg), or uncontrolled hypertension • stroke within the last 6 months. e.Renal impairment: patients with creatinine clearance less than 30 mL/min. f.Severe hepatic impairment	a.Physicians should consider the cardiovascular status of their patients. b.Physicians should consider continuous plasma tadalafil levels when evaluating the potential for interactions with medications (e.g., nitrates, α-blockers,antihypertensives and potent inhibitors of CYP3A4 and with substantial consumption of alcohol. c.Prolonged Erection d.Priapism e.Sudden loss of vision in one or both eyes: physicians should stop PDE5Is immediately. f.Sudden Hearing Loss: stop PDE5Is immediately. g.Physicians should discuss with patients the potential for tadalafil to augment the blood-pressure-lowering effect of α-blockers and antihypertensive medications h.Substantial consumption of alcohol in combination with tadalafil can increase the potential for orthostatic signs and symptoms. i.Concomitant Use of Potent Inhibitors of CYP3A4 j.Combination With Other PDE5 Inhibitors or ED therapies k.Effects on Bleeding	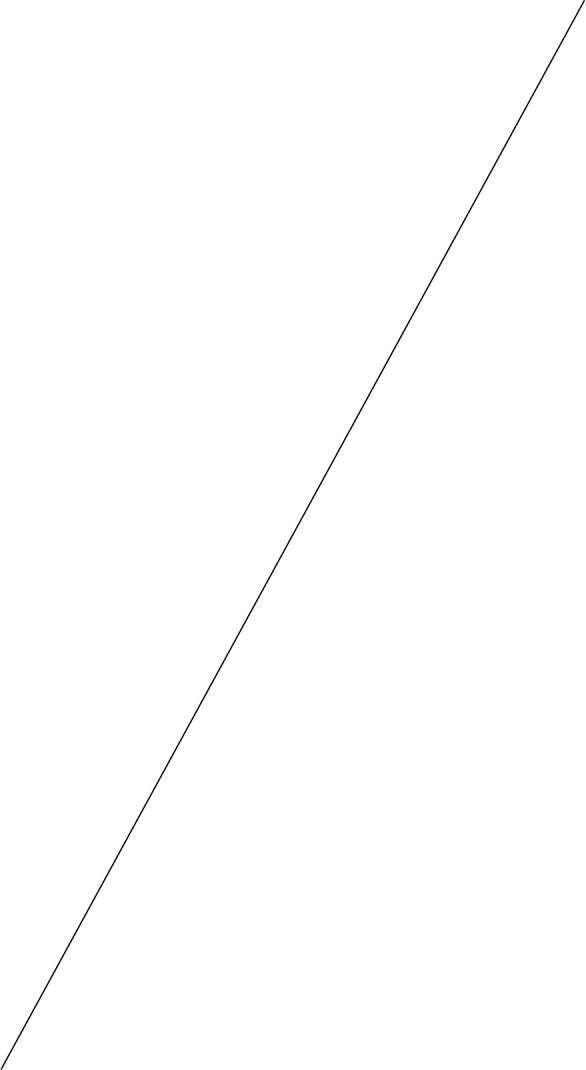

These data come from the EAU guideline (European Association of Urology, 2019) and the website of FDA. BPH, benign prostatic hyperplasia; LUTS, lower urinary tract symptoms; IFIS, intraoperative floppy iris syndrome. Orthostasis symptoms include: postural hypotension, dizziness, and vertigo. Priapism, persistent painful penile erection unrelated to sexual activity; AUR, acute urinary retention; CNS Effects, central nervous system effects including: dizziness, syncope; OAB, overactive bladder; cGMP, cyclic guanosine monophosphate; ED, erectile dysfunction; EjD, Ejaculation dysfunction; PAH, pulmonary arterial hypertension. Orthostatic signs and symptoms include: increase in heart rate, decrease in standing blood pressure, dizziness, and headache. CYP3A4, cytochrome P450 3A4; CYP2D6, cytochrome P450 2D6; PVR, post-void residual volume; 5-ARIs, 5α-reductase inhibitors; MRAs, muscarinic receptor antagonists; PDE5Is, phosphodiesterase type 5 inhibitors; Q-max, maximum urinary flow rate; QOL, quality of life; DHT, Dihydrotestosterone; PV, prostate volume; PSA, prostate-specific antigen; AUR, acute urinary retention; AUA-SI, American Urology Association Symptom Index; CCr, creatinine clearance.

## Funding

This research was supported by the National Nature Science Foundation of China (grants: 81760464 and 81860455).

## Conflict of Interest

The authors declare that the research was conducted in the absence of any commercial or financial relationships that could be construed as a potential conflict of interest.

## Abbreviations

ANCOVA, analysis of covariance; AUA, American Urological Association; AUA-SI, American Urology Association Symptom Index; AUR, Acute urinary retention; 5-ARIs, 5α-reductase inhibitors; BPH, Benign prostatic hyperplasia; BOOI, Bladder outlet obstruction index; cGMP, Cyclic guanosine monophosphate; DHT, Dihydrotestosterone; EAU, European Association of Urology; ED, Erectile dysfunction; EjD, Ejaculation dysfunction; ER, Extended-release; FDA, Food and Drug Administration; IFIS, Intraoperative floppy iris syndrome; IIEF, International Index of Erectile Function; IPSS, International Prostate Symptom Score; IR, Immediate-release; LS, Least squares; LUTS, Lower urinary tract symptoms; MRAs, Muscarinic receptor antagonists; OAB, Overactive bladder; OABSS, Overactive Bladder Symptoms Score; PCPT, Prostate Cancer Prevention Trial; PDE5Is, Phosphodiesterase type 5 inhibitors; PV, Prostate volume; PVR, Post-void residual volume; PSA, Prostate-specific antigen; PSAD, Prostate-specific antigen density; Q-max, Maximum urinary flow rate; QOL, Quality of life; RCT, Randomized controlled trial; TPV, Total prostate volume; TURP, Transurethral resection of the prostate; UI, Urge urinary incontinence.
